# Impact of Annealing Chemistry on the Properties and Performance of Microporous Annealed Particle Hydrogels

**DOI:** 10.1021/acs.biomac.4c00465

**Published:** 2024-08-27

**Authors:** Sarea Y. Recalde Phillips, Kiara D. Perez-Ponce, Elizabeth Ruben, Talia Baig, Emily Poux, Carl A. Gregory, Daniel L. Alge

**Affiliations:** † Department of Biomedical Engineering, Texas A&M University, College Station, Texas 77843, United States; ‡ Department of Chemical Engineering, Texas A&M University, College Station, Texas 77843, United States; § Department of Medical Physiology, School of Medicine, Texas A&M University, Bryan, Texas 77807, United States; ∥ Department of Materials Science and Engineering, Texas A&M University, College Station, Texas 77843, United States

## Abstract

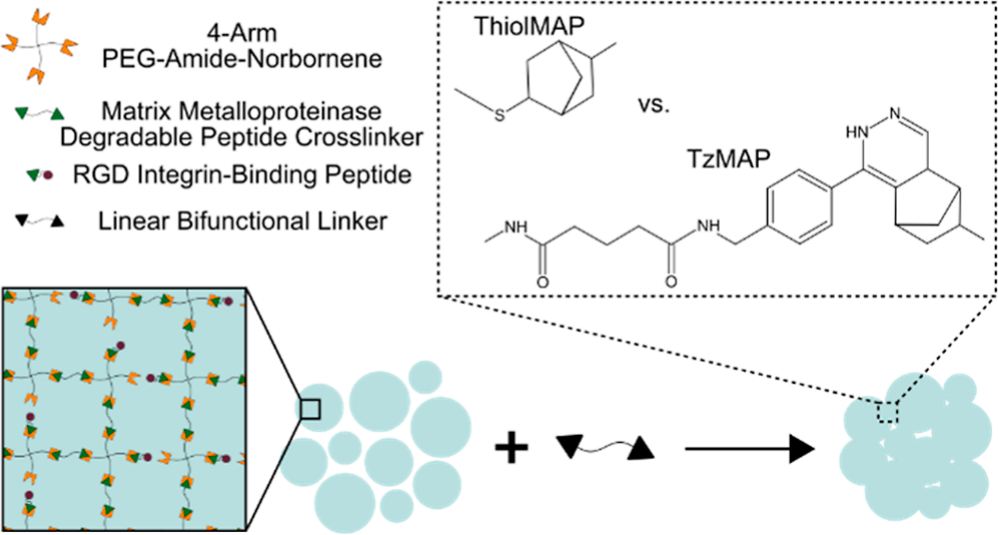

Microporous annealed particle (MAP) hydrogels are a promising class of in situ-forming scaffolds for tissue repair and regeneration. While an expansive toolkit of annealing chemistries has been described, the effects of different annealing chemistries on MAP hydrogel properties and performance have not been studied. In this study, we address this gap through a controlled head-to-head comparison of poly(ethylene glycol) (PEG)-based MAP hydrogels that were annealed using tetrazine-norbornene and thiol-norbornene click chemistry. Characterization of material properties revealed that tetrazine click annealing significantly increases MAP hydrogel shear storage modulus and results in slower in vitro degradation kinetics when microgels with a higher cross-link density are used. However, these effects are muted when the MAP hydrogels are fabricated from microgels with a lower cross-link density. In contrast, in vivo testing in murine critical-sized calvarial defects revealed that these differences in physicochemical properties do not translate to differences in bone volume or calvarial defect healing when growth-factor-loaded MAP hydrogel scaffolds are implanted into mouse calvarial defects. Nonetheless, the impact of tetrazine click annealing could be important in other applications and should be investigated further.

## 1 Introduction

Microporous annealed particle (MAP) hydrogels have recently emerged as a promising class of biomaterials with broad utility in tissue engineering. In contrast to conventional hydrogels, MAP hydrogels are granular in nature and are fabricated from microgel particles, which are physically jammed and then chemically linked together. The key advantage of MAP hydrogels is that they possess a network of highly interconnected micropores. This feature allows for increased cell infiltration and spreading, which has been shown to promote superior tissue repair and regeneration outcomes when compared to conventional nanoporous hydrogels.^1,2^ Recent studies suggest that cell infiltration and the immune response can be modulated by varying microgel size due to changes in the micropore size.^3,4^ Another important feature of MAP hydrogels is that, prior to annealing, jammed microgels exhibit shear thinning behavior.^5^ Thus, they can be injected or packed into tissue defects and then annealed in situ without compromising the formation of void spaces that promote cell infiltration and overall tissue regeneration. Importantly, MAP hydrogels have shown promising results for a myriad of tissue engineering applications, including the treatment of dermal wounds,^6^ stroke,^1^ spinal cord injury,^7,8^ volumetric muscle loss,^9^ and vocal cord injury.^10^ MAP hydrogels have also shown promise as a cell delivery platform for tissue engineering. For example, Koh et al. demonstrated enhanced cell retention and tissue regeneration when mesenchymal stem cells were subcutaneously codelivered with microgel building blocks prior to in situ assembly.^11^

An expansive toolkit of microgel synthesis and annealing strategies has been used to produce MAP hydrogels.^12−14^ Poly(ethylene glycol) (PEG), hyaluronic acid, and gelatin-based microgels have been used extensively owing to the broad popularity of these materials in tissue engineering. These materials have been formulated as microgels via fragmentation of bulk hydrogels, water-in-oil emulsions, electrospraying, microfluidics, and particle replication in nonwetting templates.^11,12,14−16^ Importantly, microgel annealing into MAP hydrogels can also be achieved through a variety of chemical reactions ranging from enzymatic cross-linking to free radical polymerizations and bioorthogonal click chemistry.^17−19^ We previously used thiol-norbornene click chemistry to anneal norbornene-functionalized PEG microgels into MAP hydrogel scaffolds and studied how variations in the physicochemical properties of the microgels influenced human mesenchymal stem cell viability and behavior.^15,20,21^ However, because this approach requires irradiation to initiate the thiol-norbornene click annealing reaction, which may not be desirable or feasible in certain clinical applications, we subsequently employed bio-orthogonal tetrazine-norbornene click chemistry.^22,23^ Tetrazine-norbornene click chemistry is widely used for bulk hydrogel synthesis,^24−30^ and it has also been used by Darling et al. to anneal MAP hydrogels.^31,32^

Recent studies have shown that secondary interactions between the dihydropyrazine cycloaddition products of tetrazine-norbornene reactions can significantly alter the hydrogel properties. We were the first to show this effect and demonstrated that PEG hydrogels cross-linked via the tetrazine-norbornene click reaction are stiffer and more resistant to degradation compared to thiol-norbornene click cross-linked PEG hydrogels.^33^ Through molecular dynamics simulations, we attributed these effects to strong secondary interactions between dihydropyrazines,^33^ and we subsequently leveraged this phenomenon to engineer supramolecular hydrogels as well as dynamic hydrogels capable of stiffening on-demand.^34^ Tetrazine click-mediated stiffening of PEG hydrogels has also been reported by Arkenberg et al., where increased stiffening and resistance to hydrolytic degradation were observed in click reactions utilizing tetrazine rather than methyltetrazine.^35−37^ Based on these prior studies, it is possible that tetrazine click annealing could alter the physicochemical properties of MAP hydrogels as well as their efficacy for tissue repair and regeneration. However, no prior studies have characterized the effects of the annealing chemistry on MAP hydrogel properties or performance.

Here, we sought to address these gaps and performed a controlled head-to-head comparison of PEG-based MAP hydrogels annealed using click chemistry. To isolate the effects of annealing chemistry, the MAP hydrogels were prepared using the same microgels, which were synthesized via off-stoichiometric thiol-norbornene click cross-linking of tetrafunctional PEG-norbornene with a dithiol peptide cross-linker. Microgels were prepared using either 5 or 20 kDa PEG-norbornene precursors and were used to evaluate dependence on microgel properties. Bifunctional PEG-dithiol and PEG-ditetrazine linkers were used to anneal these microgels into MAP hydrogels via thiol-norbornene (“ThiolMAP”) or tetrazine-norbornene (“TzMAP”) click reactions, respectively, as previously described.^15,20,22,23^ The materials were then subjected to a series of in vitro and in vivo experiments. First, the MAP hydrogel stiffness was assessed via rheology. Then, the porosity of the MAP hydrogels was evaluated via confocal fluorescence microscopy. Degradation profiles were subsequently evaluated by subjecting the MAP hydrogels to enzymatic degradation. Finally, the efficacy of the MAP hydrogels as scaffolds for bone defect regeneration was evaluated in a murine critical-sized calvarial defect model.

## 2 Materials and Methods

### 2.1 Material Synthesis

PEG–amide-tetra-norbornene (PEG–aNB) was synthesized by reacting PEG–tetra-NH_2_ (20 kDa and 5 kDa; JenKem USA) with 5-norbornene-2-carboxylic acid (Sigma-Aldrich), as previously described.^38^ PEG-diamine (3.4 kDa; Laysan Bio) was functionalized with 5-(4-(1,2,4,5-tetrazine-3-yl)-benzylamino)-5-oxopentanoic acid to synthesize PEG-ditetrazine (PEG-di-Tz), as previously described.^23,24^ Lithium acylphosphinate photoinitiator (LAP) was purchased from Sigma-Aldrich. PEG-dithiol (3.4 kDa, PEG-DT) was purchased from Laysan Bio. End-group functionalization of PEG–aNB and PEG-di-Tz was confirmed by ^1^H NMR. Cell adhesive peptide CGRGDS and matrix metalloproteinase (MMP) degradable cross-linker KCGPQGIWGQCK were synthesized with standard solid-phase Fmoc synthesis methods on a rink amide resin (Novabiochem). The peptides were purified by high pressure liquid chromatography and confirmed using matrix-assisted laser desorption-ionization time-of-flight mass spectroscopy (MALDI-TOF MS). After purification, the peptides were lyophilized and reconstituted in phosphate buffered saline (PBS, pH 7.4), and their concentrations were determined spectrophotometrically based on the absorbance at 205 nm. All prepolymer materials were reconstituted in sterile PBS, sterile filtered, subjected to endotoxin removal using Pierce high-capacity endotoxin removal spin columns (Thermo Scientific), and confirmed to be endotoxin-free through a kinetic LAL endotoxin assay (LONZA).

### 2.2 Microparticle Synthesis

PEG microgels were produced by electrospraying, as previously described.^15^ Prior to electrospraying, glassware was baked at 200 °C for 3 h to remove endotoxins,^39^ and mineral oil containing 0.40 wt % Span-80 (Tokyo Chemical Industry) was sterile-filtered prior to use. Precursor solutions with a 0.75:1 thiol:ene ratio were prepared using either 5 or 20 kDa PEG-aNB under sterile conditions (Table S1). The prepolymer solutions were loaded in a syringe fashioned with a 1 in.-long 22G blunt-tip needle. The syringe was fastened to a KDS 100 Legacy syringe pump (KD Scientific) programmed at a 12 mL/h flow rate, and the needle was submerged in mineral oil at a 16 mm needle tip-to-ring distance. The voltage used during electrospraying was set to 4 kV, and a mercury arc lamp fitted with a 365 nm filter and collimating lens (Omnicure S2000) was used to irradiate the droplets at 60 mW/cm^2^. To ensure complete curing, the microgels were irradiated for an additional 5 min after completion of the precursor solution extrusion from the syringe. Following curing, sterile PBS was added to the mineral oil/microgel mixture. The microgel-containing mixtures were then briefly vortexed and centrifuged at 4.4 × 1000 rpm for 15 min to collect microgel pellets. The microgels were then washed twice in sterile PBS, twice in 70% ethanol, and twice in sterile PBS before being suspended in sterile PBS and stored at 4 °C until use.

### 2.3 Characterization of Scaffold Properties

#### 2.3.1 MAP Scaffold Formation

Microgels were packed via centrifugation at 4.4 × 1000 rpm for 10 min prior to annealing into MAP hydrogels. Importantly, the same microgels were used for the ThiolMAP and TzMAP hydrogels. For in vitro characterization, 50 μL of packed microgels were added to 8 mm-diameter silicone molds (*V*_tot_ = 60 μL). ThiolMAPs were annealed by adding 2 mM PEG-DT and 1 mM LAP in PBS to the microgels and irradiating them with collimated 365 nm light at 10 mW/cm^2^ for 5 min. These linker and initiator concentrations were selected based on our prior work, and a light intensity of 10 mW/cm^2^ was deemed sufficient for ThiolMAP annealing when we evaluated ThiolMAP hydrogels annealed at 5, 10, and 20 mW/cm^2^ for 5 min. TzMAPs were annealed simply by adding 2 mM PEG-di-Tz in PBS and incubating at 37 °C for 1 h. Additionally, a set of microgels prepared with 5 kDa PEG-aNB but with varying concentrations of norbornene available for annealing were used to evaluate the effects of this variable on the mechanical properties of TzMAP hydrogels. Briefly, 100 μL of microgels were immersed in 600 μL of varying concentrations (0, 7, 10.5 mM) of l-cysteine (Sigma-Aldrich) and LAP and then irradiated at 10 mW/cm^2^ for 5 min. The microgel pellets were washed in PBS, centrifuged, and annealed into TzMAP hydrogels by using the aforementioned methods. Annealed MAP scaffolds were stored in PBS until they were used in characterization tests.

#### 2.3.2 Rheology

The shear storage moduli of the annealed MAP scaffolds were quantified via oscillatory shear testing on a rheometer. Testing was performed on an MCR 301 rheometer (Anton Paar) fashioned with an 8 mm-diameter parallel plate geometry. MAP scaffolds were loaded on the rheometer stand and warmed to 37 °C for the duration of data acquisition. Tests were performed at an angular frequency of 1 rad/s and 1% strain, and data was acquired every 5 s for a total of 30 data points to ensure measurement stability. These points were subsequently averaged to compute the storage moduli of individual samples.

#### 2.3.3 Porosity

The percent porosity of the ThiolMAP and TzMAP scaffolds was quantified by using confocal fluorescence microscopy. Prior to imaging, the MAP scaffolds were perfused with a 5 mg/mL aqueous solution of 155 kDa tetramethylrhodamine isothiocyanate-Dextran (Sigma-Aldrich). Dextran-perfused samples were then imaged using an SP8 Leica confocal microscope to collect 200 μm z-stacks with a 5 μm step size. The z-stacks were then analyzed in ImageJ with the Voxel Counter plugin. Percent porosity was determined by calculating 5 z-stacks per sample according to the following equation
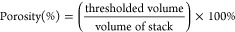


The number of pores and the cross-sectional area of pores were quantified from the same z-stacks. Z-stacks were converted to an 8 bit format on ImageJ, subjected to a minimum projection intensity on the *x*–*y* plane, thresholded, and subjected to particle analysis.

#### 2.3.4 Enzymatic Degradation

The degradation profiles of the MAP scaffolds were evaluated by exposure to collagenase since the KCGPQGIWGQCK peptide cross-linker used for microgel synthesis is derived from collagen.^40^ Collagenase B from *Clostridium histolyticum* (Sigma-Aldrich) was dissolved in a PBS solution to a final concentration of 0.1 mg/mL. MAP scaffolds were then immersed in 0.5 mL of collagenase solution and incubated at 37 °C. Sample mass was recorded in 15 min increments over 2 h. The mass at each time point (*M*_*i*_) was normalized to the initial sample mass (*M*_o_) to determine the fractional mass remaining over time using the equation below
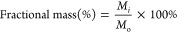


### 2.4 Calvarial Defect Surgery

Male C57BL/6 mice (Jackson Laboratory) between 13 and 19 weeks were used to evaluate the efficacy of the MAP hydrogels as scaffolds for bone defect regeneration. Housing, specimen handling, and surgical procedures described later followed guidelines established by the National Research Council’s Guide for the Care and Use of Laboratory Animals. Mice were fed and watered *ad libitum* before and after surgery.

Surgeries were conducted in accordance with an animal protocol approved by Texas A&M University’s Institutional Animal Care and Use Committee. Mice were anesthetized under 2% (v/v) isoflurane inhalant in oxygen and maintained on a heating pad at 32 °C. Once anesthetized, sustained-release buprenorphine (ZooPharm, dose = 1 mg/kg) was administered via subcutaneous injection for pain management. Depilatory cream was used to remove hair from the surgical site, which was then scrubbed with chlorhexidine (3X) and 70% isopropyl alcohol (3X). A single, longitudinal incision was made along the suture line of the mouse skull, and the skin and fascia were separated from the skull. A unilateral 2.7 mm-diameter calvarial defect was formed in the right parietal bone using osteotomy burs (Strauss Diamond), and the defect was implanted with ThiolMAPs or TzMAP scaffolds (5, 20 kDa). Microgels were swelled with 500 ng of recombinant human BMP-2 (INFUSE) prior to implantation. ThiolMAPs and TzMAPs were annealed in situ using methods and precursor solutions similar to those described previously. The total volume per implant was 6 μL (83.3 μg/mL rhBMP-2), and incisions were closed with 2–3 nylon sutures. All mice were monitored for 5 days postoperation to check for surgical complications, which were not observed during that time frame.

At 21 days and 42 days postimplantation, mice were euthanized via CO_2_ asphyxiation followed by a bilateral pneumothorax. An incision was made around the defect site and a portion of the contralateral side using a rotary blade (Strauss Diamond). A rectangular portion of the calvarium containing the defect side and an approximately equal size on the contralateral side was then removed and placed in 10% neutral buffered formalin (NBF) (Sigma-Aldrich) for 48 h. The tissue samples were then gently washed in sterile 1X PBS, placed in Carson’s fixative (10% NBF, 1.86 wt % Na_2_HPO_4_, 0.42 wt % NaOH), and stored at 4 °C until bone quantification.

### 2.5 Microcomputed Tomography Analysis

Following fixation, bone formation was quantified by microcomputed tomography (μCT) on a Skyscan 1275 scanning system (Bruker). Prior to scanning, the tissue samples were briefly washed in PBS and wrapped in parafilm. Scans were performed at a voltage of 30 kV, current of 200 μA, and pixel resolution of 18 μm in 0.5° increments over a total of 360°, and each scanned increment was based on the average of 3 frames per 0.5° rotation. 3D reconstructions were subsequently performed. Smoothing, ring artifact reduction, and beam hardening parameters were maintained at 2, 5, and 41%, respectively, for all samples. Misalignment compensation was adjusted to minimize artifacts among each sample and ranged from −6.5 to −3.0. Samples were normalized to a phantom sample’s dynamic range (max attenuation coefficient) from 0 – 0.106680. Axial images were then reconstructed and oriented for quantification by using NRecon software. Using CTAn software, image compilations of each sample were subsequently adjusted to minimum and maximum attenuation calibrations of 0.00374 and 0.09418, respectively. Bone volume and bone surface area per sample were then quantified from 101 z-slices. Finally, healing indices were calculated by normalizing the bone volume at the defect site to the contralateral side of each animal subject. Samples collected and analyzed in this study were compared to historical data of untreated defects that were also analyzed using the same μCT parameters.

### 2.6 Sample Decalcification

Following bone tissue quantification, samples were gently washed in PBS, placed in 0.5 M EDTA (pH 7.2), and stored at 4 °C for decalcification. The EDTA solution was replaced every 2–3 days until the samples became radiolucent.

### 2.7 Histology

Following decalcification, the tissue samples were dehydrated in a series of increasing ethanol concentrations (70–100% EtOH) and embedded in paraffin. Following paraffin embedding, 6 μm-thick sections were cut via microtome (Shandon Finesse 325, Thermo Fisher) and mounted on silane-treated glass slides. Tissue sections were then stained with hematoxylin (Biocare Medical) and eosin Y (Thermo Fisher) (H&E) using standard procedures or with a Masson’s trichrome kit according to the manufacturer’s protocol (StatLab). Images of stained tissues were obtained on a BioTek Lionheart FX automated microscope (Agilent).

Collagen deposition was quantified from tissue sections stained with Masson’s trichrome. Sections were collected in 50 μm increments, starting from the midsection of the defect. ImageJ was used to measure the area of collagen generated for each section, which was averaged.

### 2.8 Statistical Analysis

GraphPad Prism version 9.00 was used to perform statistical analyses on all data sets. Characterization data sets and in vivo data were analyzed using a two-way analysis of variance for annealing chemistry and microgel formulation, followed by a post hoc Tukey test for multiple comparisons. Data are presented as the average ±standard deviation.

## 3 Results

### 3.1 Microgel Synthesis and Characterization

**Figure 1:**
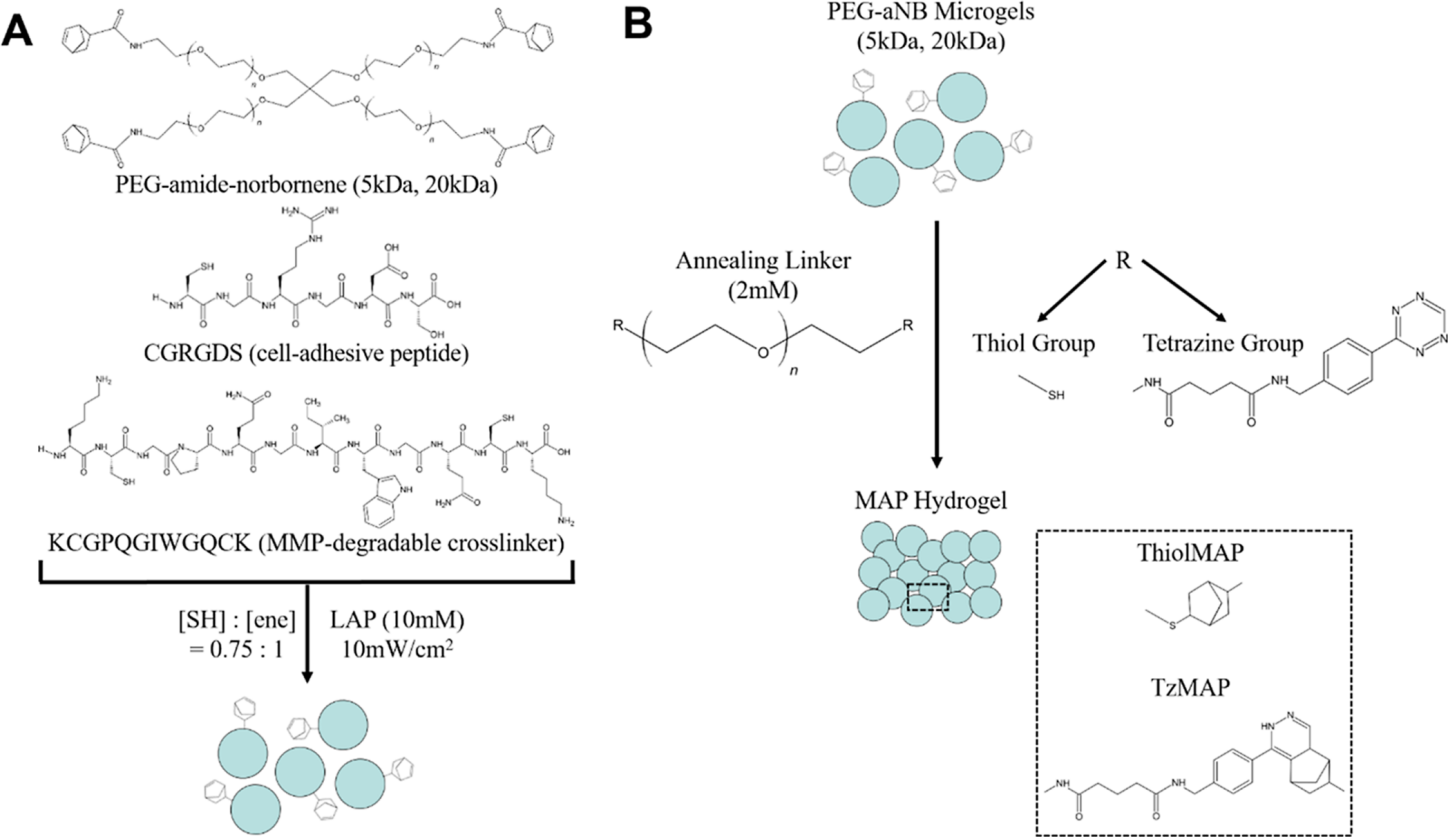
Schematic of microgel formation and MAP hydrogel assembly. (A) Microgels were formed via thiol-norbornene click chemistry and had an excess of norbornenes available for MAP hydrogel assembly. (B) Unreacted norbornenes were exploited for annealing via either thiol-norbornene or tetrazine-norbornene click reactions to form ThiolMAP and TzMAP hydrogels, respectively.

Norbornene-functionalized PEG-peptide microgels were fabricated by electrospraying and thiol-norbornene click chemistry. The microgels were cross-linked with the MMP-degradable peptide KCGPQGIWGQCK to render them degradable by cell-secreted enzymes. They were also functionalized with the integrin-binding peptide CGRGDS to permit cell adhesion. Due to the stoichiometric excess of norbornene groups in the formulations, unreacted norbornene groups were available for annealing with the appropriate linkers to form either TzMAP or ThiolMAP hydrogels (Figure 1).

**Figure 2:**
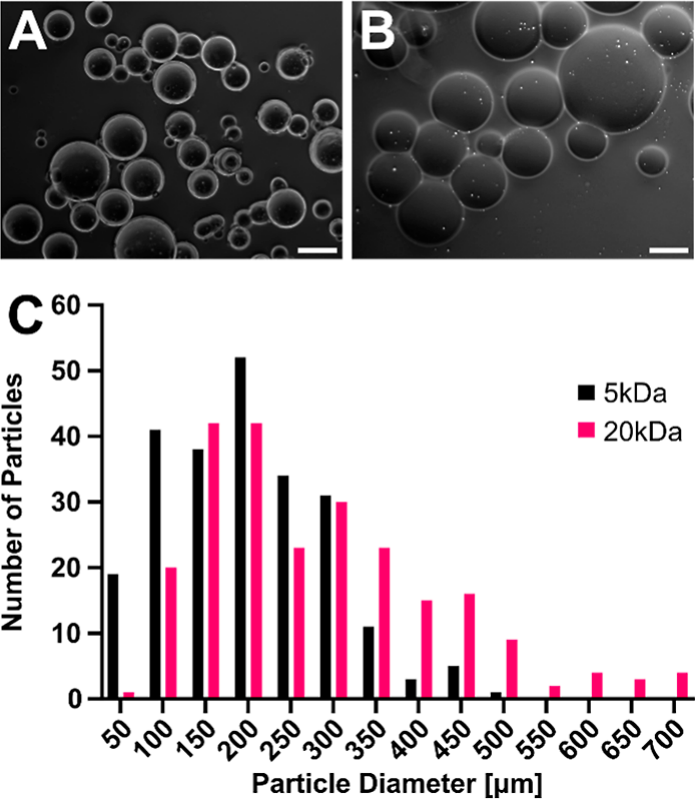
Characterization of PEG microgels. Phase-contrast images of PEG microgels prepared using (A) 5 kDa and (B) 20 kDa PEG-aNB precursors. Scale bar = 200 μm. (C) Size distribution of 5 and 20 kDa PEG microgels (*n* = 234 per group).

Image analysis revealed that microgels formed using 5 kDa PEG-aNB had smaller diameters when compared with microgels formed using 20 kDa PEG-aNB (Figure 2A, B). The diameters of these microgel formulations were 199.81 ± 95.24 and 279.31 ± 139.58 μm, respectively (Figure S2). Both microgel formulations were polydisperse, as the diameters of 5 kDa microgels ranged from approximately 45–490 μm, whereas the 20 kDa microgels ranged from approximately 70–715 μm (Figure 2C). Since droplet size depends on electrospraying parameters, which were held constant, the differences in diameter can be attributed to differences in swelling. Studies on bulk hydrogels have shown that swelling increases with increased molecular weight of PEG.^41,42^ Thus, the 5 kDa microgels are expected to be smaller. The stiffness of PEG hydrogels is also known to depend on the PEG molecular weight but follows the opposite trend. We previously showed that the mechanical properties of PEG-based microgels are comparable to bulk hydrogels formed using the same hydrogel formulations.^15^ Thus, the rheology of bulk hydrogels prepared with the same formulations as the microgels was performed to infer microgel mechanical properties. This testing revealed that the 5 and 20 kDa formulations had shear storage moduli of 9.58 ± 2.30 kPa and 2.37 ± 0.20 kPa, respectively (Figure S3).

### 3.2 MAP Hydrogel Modulus and Porosity

**Figure 3:**
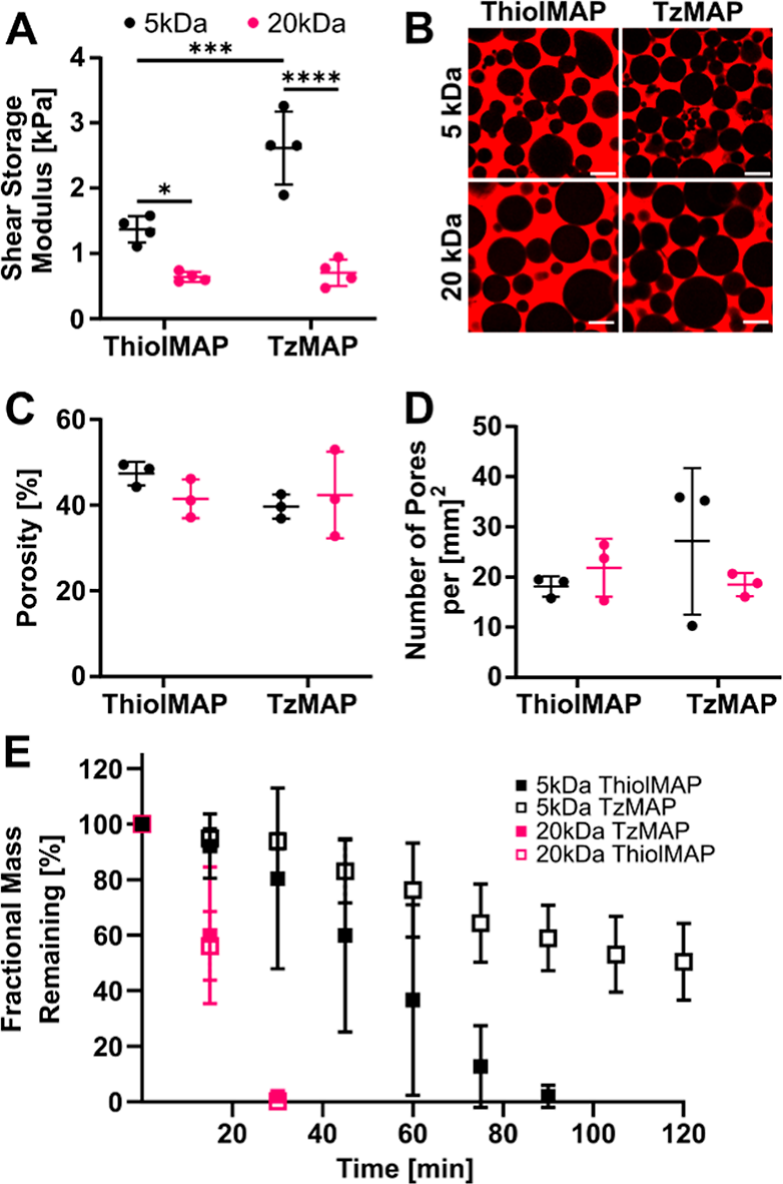
Characterization of MAP hydrogel properties. (A) Shear storage modulus of TzMAP and ThiolMAP hydrogels made using microgels synthesized using 5 and 20 kDa PEG-aNB precursors (*n* = 4). (B) Representative confocal fluorescence microscopy images of rhodamine-dextran-perfused MAP hydrogels. Scale bars = 200 μm. (C) Porosity quantification of TzMAP and ThiolMAP hydrogels (*n* = 3 hydrogels, 5 images/hydrogel). (D) Average number of pores per area in TzMAP and ThiolMAP hydrogels (*n* = 3 hydrogels, 5 images/hydrogel). (E) Degradation profile of TzMAP and ThiolMAP hydrogels (*n* = 4). Data is represented as mean ± standard deviation. * = *p* < 0.05; *** = *p* < 0.001; **** = *p* < 0.0001.

ThiolMAP and TzMAP hydrogels were assembled via thiol-norbornene and tetrazine-norbornene click reactions, respectively, using the appropriate bifunctional linkers and then subjected to a series of characterization experiments. Microgels prepared using 5 and 20 kDa precursors were used to evaluate dependence on the microgel formulation. Characterization by oscillatory shear rheology revealed that the ThiolMAP hydrogels were fully annealed after 5 min exposure to 10 mW/cm^2^ light, as increasing the light intensity did not increase the storage modulus of the ThiolMAP hydrogels (Figure S4). However, microgels prepared with 5 kDa precursors resulted in significantly stiffer MAP hydrogels when compared to their 20 kDa counterparts, as expected, regardless of the annealing chemistry used (Figure 3A). However, significant differences in storage modulus as a result of annealing chemistry were also observed, specifically for the ThiolMAP and TzMAP hydrogels prepared from 5 kDa microgels. Specifically, TzMAP hydrogels fabricated from 5 kDa microgels had a storage modulus of 2.62 ± 0.56 kPa, which was a statistically significant 1.91-fold increase compared to the 1.37 ± 0.20 kPa measured for the ThiolMAP hydrogels fabricated from the same 5 kDa microgels (*p* = 0.0006). In contrast to the stark differences in modulus observed between 5 kDa ThiolMAP and TzMAP hydrogel groups, annealing chemistry did not have a significant effect on the modulus of MAP hydrogels fabricated from 20 kDa microgels, as the TzMAP and ThiolMAP groups had shear storage moduli of 0.70 ± 0.20 and 0.64 ± 0.08 kPa, respectively (*p* = 0.9919). Interestingly, capping norbornene groups in the 5 kDa microgels by prereacting them with 7 mM and 10.5 mM l-cysteine prior to TzMAP hydrogel formation reduced the shear storage modulus by approximately 50% (Figure S5).

Effects on the MAP hydrogel porosity were less pronounced. Porosity was evaluated using confocal microscopy following perfusion with rhodamine-labeled dextran (Figure 3B). However, there were negligible differences in overall porosity between groups; percent porosity measurements for TzMAP hydrogels fabricated from 20 kDa and 5 kDa microgel formulations were 42.4 ± 10.1 and 39.7 ± 2.80%, respectively, whereas they were 41.5 ± 4.50 and 47.4 ± 2.75%, respectively, for ThiolMAP hydrogels (Figure 3C). None of the differences was statistically significant. Interestingly, clusters of smaller-diameter microgels appeared to aggregate and generate smaller, fragmented void spaces more frequently among the 5 and 20 kDa TzMAP hydrogels than their ThiolMAP counterparts, but there was only a marginal increase in the number of pores observed in 5 kDa TzMAP hydrogels when compared to their 20 kDa counterparts (Figure 3D). Differences between pore size area of 5 kDa ThiolMAP and TzMAP hydrogel groups were statistically significant (2.69 × 10^3^ ± 6.73 × 10^3^ vs 1.63 × 10^3^ ± 5.21 × 10^3^ μm^2^; *p* = 0.0032) (Figure S6). Average pore sizes of 20 kDa ThiolMAP and TzMAP hydrogels, however, were statistically insignificant (2.32 × 10^3^ ± 8.23 × 10^3^ vs 1.31 × 10^3^ ± 1.90 × 10^3^ μm^2^; *p* = 0.1284).

### 3.3 MAP Hydrogel Degradability

The microgels used to assemble the MAP hydrogels were formed with an MMP-degradable cross-linker, thereby providing a site for enzymatic degradation.^43,44^ Thus, the effects of annealing chemistry on the MAP hydrogel degradation profile were assessed in vitro for ThiolMAPs and TzMAPs (Figure 3E). The ThiolMAP and TzMAP hydrogels formed from the less tightly cross-linked 20 kDa microgels degraded quickly and exhibited drastic decreases in mass in the first 15 min of incubation in the collagenase solution. Moreover, these MAP hydrogel groups degraded completely by 45 min, and there was no apparent difference in degradation rate due to the annealing chemistry. In contrast, annealing chemistry was observed to affect the degradation rate when comparing ThiolMAP and TzMAP hydrogels fabricated from more tightly cross-linked 5 kDa microgels. The 5 kDa ThiolMAP hydrogels completely degraded by 105 min, and the 5 kDa TzMAP counterpart exhibited an extended degradation profile and retained 50.4 ± 13.9% of its initial mass at the end of the experiment despite being fabricated from the same microgels. Overall, these data mirror the effects of the annealing chemistry on the MAP hydrogel stiffness.

### 3.4 Bone Defect Regeneration after MAP Hydrogel Implantation

**Figure 4:**
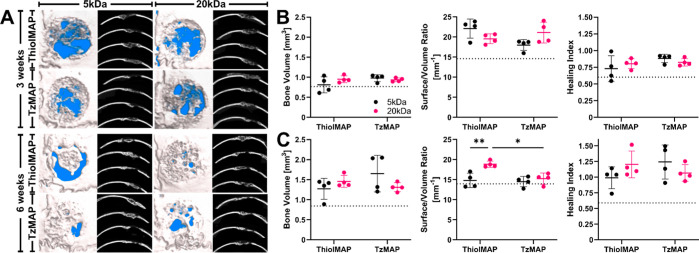
μCT analysis of calvarial bone defect regeneration after treatment with TzMAP and ThiolMAP hydrogels. (A) 3 week (upper) and 6 week (lower) 3D reconstructions and axial images of mouse calvaria implanted with BMP-2 loaded TzMAP and ThiolMAP hydrogels. The background of the 3D reconstructions was arbitrarily set to blue to provide visual contrast between the background and the defect site. Bone volume, S/V ratio, and healing index of MAP hydrogels (B) 3 weeks and (C) 6 weeks postimplantation (*n* = 4). Dashed lines represent measurements for untreated defects. Data is represented as mean ± standard deviation. * = *p* < 0.05; ** = *p* < 0.01.

To evaluate the effects of annealing chemistry on MAP hydrogel efficacy for tissue regeneration, TzMAP and ThiolMAP hydrogels were implanted into murine critical-sized calvarial defects. To stimulate bone regeneration, the MAP hydrogels were loaded with 500 ng of rhBMP-2, which is well documented to promote bone formation^45,46^ and is FDA approved for clinical applications.^47,48^ No adverse events were observed immediately after MAP hydrogel implantation or for the duration of the 3 and 6 week time points. Tissue samples were first evaluated using μCT, with 3D reconstructions and quantitative analysis being performed at 3 and 6 weeks postimplantation (Figures 4A and S7).

Bone formation was observed at 3 weeks in all treatment groups. In general, larger and denser bone fragments were prominent around the upper and lower portions of the MAP hydrogels, while smaller bone fragments were observed in the pores of the MAP hydrogels. Quantitative analysis revealed similar levels of bone formation and only minor differences between the treatment groups (Figure 4B). There were negligible differences in terms of the volume of bone formed within the defects. TzMAP and ThiolMAP hydrogels formed using 5 kDa microgels resulted in 0.98 ± 0.09 and 0.81 ± 0.20 mm^3^ of bone, respectively, whereas TzMAP and ThiolMAP hydrogels formed from 20 kDa microgels resulted in 0.94 ± 0.05 and 0.95 ± 0.09 mm^3^, respectively. The effects of annealing chemistry and microgel formulation on bone volume were not statistically significant, but all treatment groups resulted in higher bone volumes than the untreated group, which had a volume of 0.76 ± 0.17 mm^3^. Surface area-to-volume (S/V) ratio measurements revealed minor differences between ThiolMAP and TzMAP hydrogels, and all treatment groups had higher S/V ratios than did untreated defects, which had an S/V ratio of 14.6 ± 1.51 mm^–1^. The 5 kDa ThiolMAP hydrogels had a marginally higher S/V ratio than their TzMAP counterpart (22.1 ± 2.37 vs 18.0 ± 1.37 mm^–1^; *p* = 0.06), suggesting more bone fragments within the defect. However, the S/V ratios of the 20 kDa TzMAP and ThiolMAP hydrogels (21.1 ± 2.57 vs 19.54 ± 1.22 mm^–1^; *p* = 0.69) were comparable and similar to the 5 kDaTzMAP group. Healing indices at 3 weeks for the 20 kDa TzMAP and ThiolMAP groups were 0.825 ± 0.06 and 0.80 ± 0.08, respectively, whereas the 5 kDa TzMAP and ThiolMAP groups resulted in healing indices of 0.88 ± 0.06 and 0.73 ± 0.19, respectively. All treatment groups resulted in higher healing index values than did the untreated group (0.60 ± 0.15), but the differences between groups were not statistically significant.

Bone formation increased by 6 weeks (Figure 4C). More bone nodules were apparent in the MAP hydrogel pores, and increased interconnectivity of bone throughout the hydrogel implants was apparent for all hydrogel groups. Bone volume measurements confirmed higher levels than at 3 weeks. TzMAP and ThiolMAP hydrogels formed using microgels from the 5 kDa formulation resulted in 1.65 ± 0.45 and 1.28 ± 0.26 mm^3^ of bone, respectively, whereas TzMAP and ThiolMAP hydrogels formed from 20 kDa microgels resulted in 1.31 ± 0.11 and 1.46 ± 0.15 mm^3^, respectively. The volume of bone formation was substantially higher than that for untreated defects (0.84 ± 0.26 mm^3^) but was comparable among the four treatment groups. The S/V ratios decreased for all treatment groups compared to 3 weeks, indicating that there were fewer bone fragments and more continuous bone deposition networks throughout the defects. Minor differences in S/V ratios were observed. The stiffer 5 kDa ThiolMAP hydrogels demonstrated a significantly lower S/V ratio than their 20 kDa counterpart (14.8 ± 1.81 vs 18.9 ± 0.79 mm^–1^; *p* = 0.006), and the average S/V ratio for the 20 kDa ThiolMAP group was 1.27 times higher than its 5 kDa counterpart. TzMAP hydrogels from the 20 kDa and 5 kDa groups had comparable S/V ratios (15.2 ± 1.40 vs 14.5 ± 1.28 mm^–1^, respectively), and 20 kDa TzMAP hydrogels had a significantly lower S/V ratio than their 20 kDa ThiolMAP counterparts (*p* = 0.01). Healing indices were comparable among treatment groups and ranged from 0.99–1.24, while untreated defects had a healing index of 0.59 ± 0.12. Notably, however, spherical voids were visible in the 3D reconstruction and axial images, indicating remnant portions of the MAP hydrogels that had not yet degraded.

**Figure 5:**
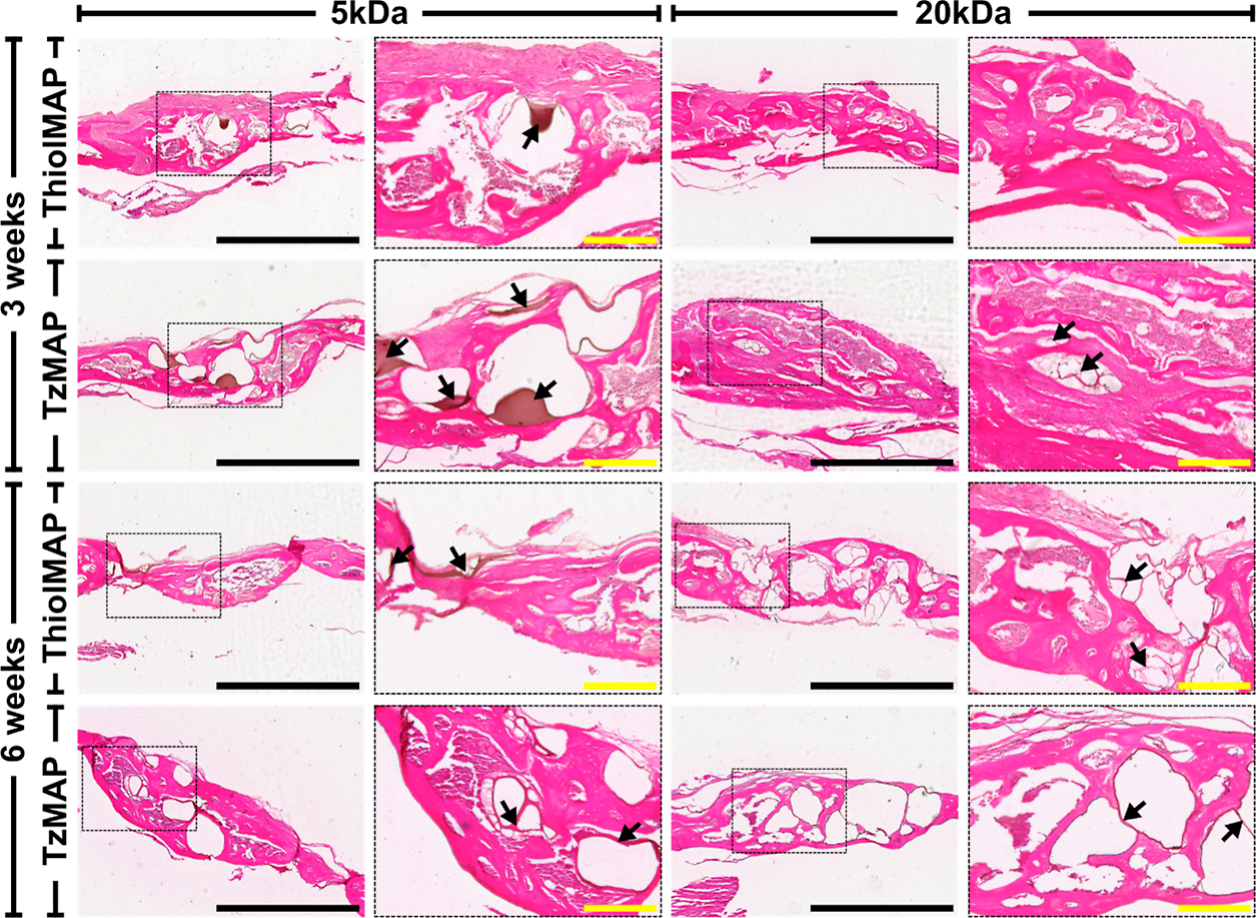
H&E staining of calvarial defects 3 and 6 weeks after implantation of TzMAP and ThiolMAP hydrogels. Defect sites were magnified at 4× (scale bar = 1 mm). Areas of the defect sites encased in the dashed boxes were further magnified at 10× (scale bar = 200 μm). Arrows denote visible fragments of hydrogel material remaining at the defect site.

**Figure 6:**
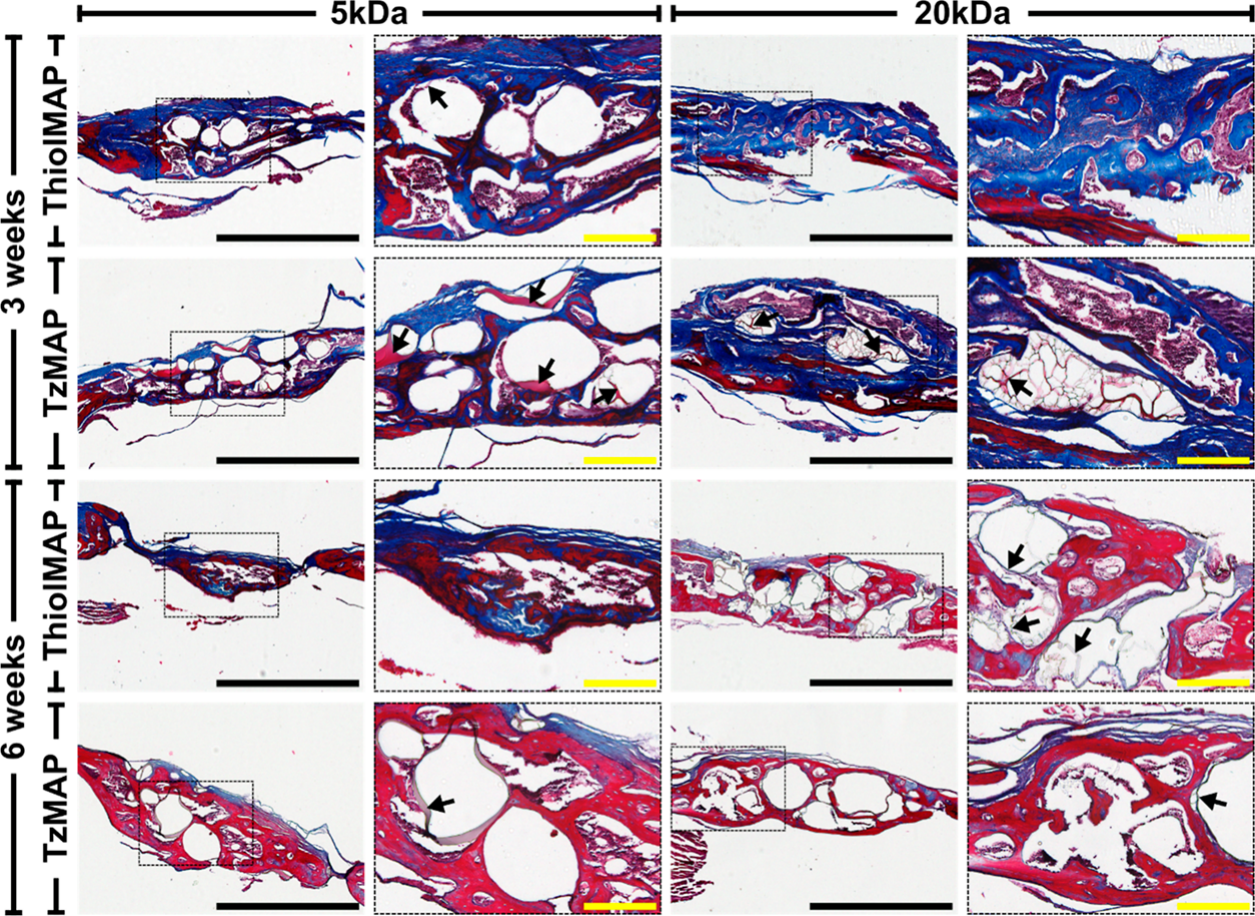
Masson’s Trichrome staining of calvarial defects 3 and 6 weeks after implantation of TzMAP and ThiolMAP hydrogels. Defect sites were magnified at 4× (scale bar = 1 mm). Areas of the defect sites encased in the dashed boxes were further magnified at 10× (scale bar = 200 μm). Arrows denote visible fragments of hydrogel material remaining at the defect site.

Histological analysis corroborated the μCT findings and provided additional insight on bone formation. H&E staining revealed a high degree of cell infiltration into the MAP scaffolds for all treatment groups (Figure 5). Cells and the deposited matrix were observed throughout the micropores of the MAP hydrogels, and the capacity for cells to infiltrate the interconnected spaces was not impeded by microgel type or annealing chemistry. Remnants of the microgels were apparent at the 3 week time point and persisted to the 6 week time point, which agreed with the μCT observations. Masson’s trichrome staining (Figure 6) also showed numerous cells around individual microgels and within the void spaces of the MAP hydrogels. At 3 weeks, collagen matrix deposition was observed throughout the defect sites implanted with both TzMAP and ThiolMAP hydrogels. The area of collagen deposition at 3 weeks was comparable between TzMAP and ThiolMAP hydrogels assembled with either the 20 or 5 kDa microgel formulations, and this trend persisted at 6 weeks (Figure S8). At 3 weeks, the tissues had an appearance consistent with immature bone and did not appear to vary between the treatment groups. By 6 weeks, mature bone had formed, as depicted by the red staining found at the center of the defect sites. No differences were observed between the treatment groups.

## 4 Discussion

MAP hydrogels are an attractive platform for tissue regeneration due to their pore interconnectivity and tunable physicochemical properties that promote cell infiltration and improved regenerative outcomes. Tetrazine click chemistry is an attractive approach for in situ MAP scaffold annealing within tissue defects. Unlike thiol–ene click chemistry, which requires an initiator for the reaction to proceed, this bio-orthogonal click reaction proceeds spontaneously under physiological conditions without requiring an initiator, catalyst, or irradiation.^49,50^ However, prior studies have shown that secondary interactions between the dihydropyrazine cycloaddition products of tetrazine-norbornene click reactions can significantly affect the properties of bulk hydrogels,^34^ but the extent to which MAP hydrogel properties are influenced and any resulting impacts on in vivo performance for tissue repair and regeneration have not been studied. To address this gap, we exploited the reactivity of norbornene to anneal MAP hydrogels using tetrazine-norbornene and thiol-norbornene click chemistry (i.e., TzMAP and ThiolMAP hydrogels, respectively) and performed a head-to-head comparison of these annealing chemistries. To isolate the effects of annealing chemistry, TzMAP and ThiolMAP hydrogels were formed by using the same norbornene-functionalized microgel building blocks. Microgels with relatively low and high cross-link densities were fabricated from 20 and 5 kDa PEG-aNB precursors, respectively, to investigate dependence on microgel formulation.

Characterization of material properties revealed that the cross-linking density of the microgel building blocks significantly affected MAP hydrogel properties. As expected, using a 5 kDa precursor to produce microgels resulted in MAP hydrogels that were stiffer due to their higher cross-link density, irrespective of annealing chemistry (Figure 3). MAP hydrogels produced from these 5 kDa microgels also degraded more slowly. This trend was expected since the enzymatic degradation rate has been shown to increase in response to the increased molecular weight of the PEG used during hydrogel formation.^51^ Importantly, however, the effects of microgel formulation were more pronounced in the TzMAP hydrogels, as the TzMAP hydrogels produced with 5 kDa microgels had a markedly increased storage modulus compared to the other MAP hydrogel formulations and did not degrade entirely over the experimental time frame. A previous head-to-head comparison of thiol-norbornene and tetrazine-norbornene click cross-linked bulk PEG hydrogels by Holt et al. reported similar results, which were attributed to secondary interactions between the dihydropyrazine products of the tetrazine-norbornene click reaction.^33^ Our findings suggest that the same phenomenon impacts MAP hydrogels, likely because the linker molecules used for annealing are able to diffuse into the microgels. Importantly, however, the effects were muted for MAP hydrogels formed using lower cross-link density 20 kDa microgels. Had the 20 kDa microgels been formed with a higher working concentration of PEG-aNB and subsequently a higher concentration of norbornene groups available for TNCP formation, it could be posited that differences in stiffness and degradation between 20 kDa TzMAP and ThiolMAP hydrogels would be more pronounced. Regardless, this finding sheds light on the interplay between microgel formulation and the local density of click products formed during assembly and how these factors impact the influence of annealing chemistry on MAP hydrogel properties.

Another feature examined in our study was the porosity of the assembled MAP hydrogels. Microporosity is an important feature of MAP hydrogels, and it has been shown that cell spreading and proliferation are reduced as pore size decreases.^32,52,53^ Due to the potential for secondary interactions between tetrazine-norbornene click products,^33−35^ we hypothesized that annealing chemistry could affect microgel–microgel interactions and influence the pore structure in the MAP hydrogels. Interestingly, we found differences in the number of pores and range of pore sizes as a result of annealing chemistry. A marginal increase in the number of pores formed was observed in 5 kDa TzMAP hydrogels when compared to their ThiolMAP counterpart, and median pore size was significantly lower in the former group when compared to the latter group. These results highlight annealing chemistry, particularly tetrazine click annealing chemistry, as a variable that can potentially influence the inner porosity of MAP hydrogels.

Interestingly, despite the differences in physicochemical properties, annealing chemistry had no apparent effect on bone regeneration when the MAP hydrogel scaffolds were loaded with rhBMP-2 and implanted into mouse calvarial defects. Rather, owing to the interconnected microporosity, cells were able to effectively infiltrate the TzMAP and ThiolMAP hydrogel scaffolds and deposit a significantly increased amount of mineralized tissue compared to that with no treatment. This observation is consistent with prior studies demonstrating effective cell infiltration and tissue regeneration with MAP hydrogels in other applications.^2,10,16^ Moreover, the annealing chemistry did not significantly affect the volume of bone formed within the defects or the healing index, regardless of the microgel stiffness, and the calvarial defects were almost completely filled with bone after 6 weeks. This result suggests that the extended degradation profile of the TzMAP hydrogels formed from the 5 kDa microgel formulation does not impact bone regeneration, at least in the context of rhMP-2 delivery into calvarial defects. In fact, our results support the use of either ThiolMAP or TzMAP hydrogels in inducing mature bone formation and overall improved regenerative outcomes in a critical-size defect. This finding is important considering that tetrazine click chemistry offers a facile approach to in situ annealing of MAP hydrogels with bio-orthogonal chemistry and would eliminate the use of initiators necessary for MAP hydrogels assembled with thiol–ene click chemistry.

A limitation of our study is that the findings on the effects of tetrazine click annealing may be limited to the specific tetrazine-functionalized linker that we used. Our linker was functionalized with a 1,2,4,5-tetrazine with a hydrogen atom in the 6-position, which was chosen because of the fast reaction kinetics of this tetrazine structure with norbornene.^54^ While methyl tetrazines exhibit slower reaction kinetics, evidence suggests that they may not exhibit the same propensity for secondary interactions.^35^ Thus, methyl tetrazine-functionalized linkers could potentially be employed to mitigate undesired effects on physicochemical properties. Additionally, the effects of microgel size are unclear since we fabricated the MAP hydrogels from polydisperse electrosprayed microgels, and the effects of annealing chemistry on MAP hydrogels made from monodisperse microgel populations should be studied in the future. The effect of the norbornene density is also unclear. The 5 kDa microgel formulation had many more norbornene groups available for annealing, and capping these norbornenes with cysteine significantly reduced the TzMAP hydrogel modulus. Future studies should further elucidate the relationship between functional group density and the impact of secondary interactions induced from tetrazine-norbornene click products on the final MAP material properties.

## 5 Conclusions

In this study, we performed head-to-head analyses of TzMAP and ThiolMAP hydrogels to evaluate the effects of annealing chemistry on MAP hydrogel properties and performance. In vitro testing revealed distinct modulation of MAP hydrogel properties as a result of annealing chemistry, as TzMAP hydrogels made from microgels with a higher cross-link density were stiffer and more resistant to degradation than their ThiolMAP counterparts. Despite the differences in material properties observed in vitro, osteogenic efficacy was not affected when these MAP hydrogels were used to deliver rhBMP-2 to murine calvarial defects. Rather, extensive bone formation and nearly complete defect regeneration were observed at 6 weeks post-treatment, regardless of the microgels or annealing chemistry used. This result suggests that, at least in this application, TzMAP hydrogels effectively facilitate regeneration despite the effects of tetrazine click annealing on MAP hydrogel properties. However, it is possible that the in vivo performance in other applications could be impacted. Thus, future studies should investigate this possibility as well as the impact of alternative tetrazine linkers.

## Data Availability

Data will be made available on request.

## Supporting Information Available

^1^H NMR spectrum of modified PEG; precursor formulations of electrosprayed PEG microgels; size analysis of PEG microgels; rheology of bulk PEG hydrogels; rheology of ThiolMAP hydrogels assembled with varying light intensity; shear modulus change as a result of capping norbornenes available for cross-linking; pore analysis of MAP hydrogels; 3D reconstructions of calvarial samples; and quantification of in vivo collagen deposition at defect sites (PDF)

## References

[ref1] Nih L. R.; Sideris E.; Carmichael S. T.; Segura T. Injection of Microporous Annealing Particle (MAP) Hydrogels in the Stroke Cavity Reduces Gliosis and Inflammation and Promotes NPC Migration to the Lesion. Adv. Mater. 2017, 29 (32), 1606471 10.1002/adma.201606471. PMC5595584 28650574

[ref2] Griffin D. R.; Weaver W. M.; Scumpia P. O.; Di Carlo D.; Segura T. Accelerated Wound Healing by Injectable Microporous Gel Scaffolds Assembled from Annealed Building Blocks. Nat. Mater. 2015, 14 (7), 737–744. 10.1038/nmat4294. 26030305 PMC4615579

[ref3] Liu Y.; Suarez-Arnedo A.; Caston E. L. P.; Riley L.; Schneider M.; Segura T. Exploring the Role of Spatial Confinement in Immune Cell Recruitment and Regeneration of Skin Wounds. Adv. Mater. 2023, 35 (49), 2304049 10.1002/adma.202304049. PMC10874253 37721722

[ref4] Lowen J. M.; Bond G. C.; Griffin K. H.; Shimamoto N. K.; Thai V. L.; Leach J. K. Multisized Photoannealable Microgels Regulate Cell Spreading, Aggregation, and Macrophage Phenotype through Microporous Void Space. Adv. Healthcare Mater. 2023, 12 (13), 2202239 10.1002/adhm.202202239. PMC10198868 36719946

[ref5] Emiroglu D. B.; Bekcic A.; Dranseike D.; Zhang X.; Zambelli T.; DeMello A. J.; Tibbitt M. W. Building Block Properties Govern Granular Hydrogel Mechanics through Contact Deformations. Sci. Adv. 2022, 8 (50), eadd8570 10.1126/sciadv.add8570. 36525484 PMC9757745

[ref6] Pruett L.; Ellis R.; McDermott M.; Roosa C.; Griffin D. Spatially Heterogeneous Epidermal Growth Factor Release from Microporous Annealed Particle (MAP) Hydrogel for Improved Wound Closure. J. Mater. Chem. B 2021, 9 (35), 7132–7139. 10.1039/D1TB00715G. 33998629 PMC8446298

[ref7] Ross B. C.; Kent R. N. III; Saunders M. N.; Schwartz S. R.; Smiley B. M.; Hocevar S. E.; Chen S.-C.; Xiao C.; Williams L. A.; Anderson A. J.; Cummings B. J.; Baker B. M.; Shea L. D. Building-Block Size Mediates Microporous Annealed Particle Hydrogel Tube Microenvironment Following Spinal Cord Injury. Adv. Healthcare Mater. 2023, 2302498 10.1002/adhm.202302498. PMC10972780 37768019

[ref8] Ehsanipour A.; Sathialingam M.; Rad L. M.; de Rutte J.; Bierman R. D.; Liang J.; Xiao W.; Di Carlo D.; Seidlits S. K. Injectable, Macroporous Scaffolds for Delivery of Therapeutic Genes to the Injured Spinal Cord. APL Bioeng. 2021, 5 (1), 16104 10.1063/5.0035291. PMC7946441 33728392

[ref9] Basurto I. M.; Passipieri J. A.; Gardner G. M.; Smith K. K.; Amacher A. R.; Hansrisuk A. I.; Christ G. J.; Caliari S. R. Photoreactive Hydrogel Stiffness Influences Volumetric Muscle Loss Repair. Tissue Eng., Part A 2022, 28 (7–8), 312–329. 10.1089/ten.tea.2021.0137. 34409861 PMC9057873

[ref10] Pruett L. J.; Kenny H. L.; Swift W. M.; Catallo K. J.; Apsel Z. R.; Salopek L. S.; Scumpia P. O.; Cottler P. S.; Griffin D. R.; Daniero J. J. De Novo Tissue Formation Using Custom Microporous Annealed Particle Hydrogel Provides Long-Term Vocal Fold Augmentation. npj Regen. Med. 2023, 8 (1), 10 10.1038/s41536-023-00281-8. 36823180 PMC9950481

[ref11] Koh J.; Griffin D. R.; Archang M. M.; Feng A.-C.; Horn T.; Margolis M.; Zalazar D.; Segura T.; Scumpia P. O.; Di Carlo D. Enhanced In Vivo Delivery of Stem Cells Using Microporous Annealed Particle Scaffolds. Small 2019, 15 (39), e1903147 10.1002/smll.201903147. 31410986 PMC6761037

[ref12] Muir V. G.; Qazi T. H.; Shan J.; Groll J.; Burdick J. A. Influence of Microgel Fabrication Technique on Granular Hydrogel Properties. ACS Biomater. Sci. Eng. 2021, 7 (9), 4269–4281. 10.1021/acsbiomaterials.0c01612. 33591726 PMC8966052

[ref13] Du Y.; Lo E.; Ali S.; Khademhosseini A. Directed Assembly of Cell-Laden Microgels for Fabrication of 3D Tissue Constructs. Proc. Natl. Acad. Sci. U.S.A. 2008, 105 (28), 9522–9527. 10.1073/pnas.0801866105. 18599452 PMC2474514

[ref14] Widener A. E.; Duraivel S.; Angelini T. E.; Phelps E. A. Injectable Microporous Annealed Particle Hydrogel Based on Guest–Host-Interlinked Polyethylene Glycol Maleimide Microgels. Adv. NanoBiomed Res. 2022, 2 (10), 2200030 10.1002/anbr.202200030. 36419640 PMC9678130

[ref15] Xin S.; Wyman O. M.; Alge D. L. Assembly of PEG Microgels into Porous Cell-Instructive 3D Scaffolds via Thiol-Ene Click Chemistry. Adv. Healthcare Mater. 2018, 7 (11), e1800160 10.1002/adhm.201800160. PMC6262827 29663702

[ref16] Suturin A. C.; Krüger A. J. D.; Neidig K.; Klos N.; Dolfen N.; Bund M.; Gronemann T.; Sebers R.; Manukanc A.; Yazdani G.; Kittel Y.; Rommel D.; Haraszti T.; Köhler J.; De Laporte L. Annealing High Aspect Ratio Microgels into Macroporous 3D Scaffolds Allows for Higher Porosities and Effective Cell Migration. Adv. Healthcare Mater. 2022, 11 (24), e2200989 10.1002/adhm.202200989. PMC11469137 36100464

[ref17] Sideris E.; Griffin D. R.; Ding Y.; Li S.; Weaver W. M.; Di Carlo D.; Hsiai T.; Segura T. Particle Hydrogels Based on Hyaluronic Acid Building Blocks. ACS Biomater. Sci. Eng. 2016, 2 (11), 2034–2041. 10.1021/acsbiomaterials.6b00444. 33440539

[ref18] Pfaff B. N.; Pruett L. J.; Cornell N. J.; de Rutte J.; Di Carlo D.; Highley C. B.; Griffin D. R. Selective and Improved Photoannealing of Microporous Annealed Particle (MAP) Scaffolds. ACS Biomater. Sci. Eng. 2021, 7 (2), 422–427. 10.1021/acsbiomaterials.0c01580. 33423459 PMC8408836

[ref19] Coronel M. M.; Martin K. E.; Hunckler M. D.; Kalelkar P.; Shah R. M.; García A. J. Hydrolytically Degradable Microgels with Tunable Mechanical Properties Modulate the Host Immune Response. Small 2022, 18 (36), e2106896 10.1002/smll.202106896. 35274457 PMC10288386

[ref20] Xin S.; Gregory C. A.; Alge D. L. Interplay between Degradability and Integrin Signaling on Mesenchymal Stem Cell Function within Poly(Ethylene Glycol) Based Microporous Annealed Particle Hydrogels. Acta Biomater. 2020, 101, 227–236. 10.1016/j.actbio.2019.11.009. 31711899 PMC6960331

[ref21] Xin S.; Dai J.; Gregory C. A.; Han A.; Alge D. L. Creating Physicochemical Gradients in Modular Microporous Annealed Particle Hydrogels via a Microfluidic Method. Adv. Funct. Mater. 2020, 30 (6), 1907102 10.1002/adfm.201907102. 38213754 PMC10783553

[ref22] Isaac A.; Jivan F.; Xin S.; Hardin J.; Luan X.; Pandya M.; Diekwisch T. G. H.; Alge D. L. Microporous Bio-Orthogonally Annealed Particle Hydrogels for Tissue Engineering and Regenerative Medicine. ACS Biomater. Sci. Eng. 2019, 5 (12), 6395–6404. 10.1021/acsbiomaterials.9b01205. 33417792 PMC7992163

[ref23] Jivan F.; Alge D. L. Bio-Orthogonal, Site-Selective Conjugation of Recombinant Proteins to Microporous Annealed Particle Hydrogels for Tissue Engineering. Adv. Ther. 2020, 3 (1), 1900148 10.1002/adtp.201900148. PMC11178337 38882245

[ref24] Alge D. L.; Azagarsamy M. A.; Donohue D. F.; Anseth K. S. Synthetically Tractable Click Hydrogels for Three-Dimensional Cell Culture Formed Using Tetrazine–Norbornene Chemistry. Biomacromolecules 2013, 14 (4), 949–953. 10.1021/bm4000508. 23448682 PMC3623454

[ref25] Contessi Negrini N.; Angelova Volponi A.; Sharpe P. T.; Celiz A. D. Tunable Cross-Linking and Adhesion of Gelatin Hydrogels via Bioorthogonal Click Chemistry. ACS Biomater. Sci. Eng. 2021, 7 (9), 4330–4346. 10.1021/acsbiomaterials.1c00136. 34086456

[ref26] Gultian K. A.; Gandhi R.; Kim T. W. B.; Vega S. L. Self-Forming Norbornene-Tetrazine Hydrogels with Independently Tunable Properties. Macromol. Biosci. 2023, 23 (3), e2200425 10.1002/mabi.202200425. 36493315 PMC10023368

[ref27] Desai R. M.; Koshy S. T.; Hilderbrand S. A.; Mooney D. J.; Joshi N. S. Versatile Click Alginate Hydrogels Crosslinked via Tetrazine–Norbornene Chemistry. Biomaterials 2015, 50, 30–37. 10.1016/j.biomaterials.2015.01.048. 25736493

[ref28] Zhang Z.; He C.; Chen X. Injectable Click Polypeptide Hydrogels via Tetrazine-Norbornene Chemistry for Localized Cisplatin Release. Polymers (Basel) 2020, 12 (4), 884 10.3390/polym12040884. 32290336 PMC7240560

[ref29] Famili A.; Rajagopal K. Bio-Orthogonal Cross-Linking Chemistry Enables In Situ Protein Encapsulation and Provides Sustained Release from Hyaluronic Acid Based Hydrogels. Mol. Pharmaceutics 2017, 14 (6), 1961–1968. 10.1021/acs.molpharmaceut.7b00067. 28463007

[ref30] Ziegler C. E.; Graf M.; Nagaoka M.; Lehr H.; Goepferich A. M. In Situ Forming IEDDA Hydrogels with Tunable Gelation Time Release High-Molecular Weight Proteins in a Controlled Manner over an Extended Time. Biomacromolecules 2021, 22 (8), 3223–3236. 10.1021/acs.biomac.1c00299. 34270216

[ref31] Darling N. J.; Xi W.; Sideris E.; Anderson A. R.; Pong C.; Carmichael S. T.; Segura T. Click by Click Microporous Annealed Particle (MAP) Scaffolds. Adv. Healthcare Mater. 2020, 9 (10), 1901391 10.1002/adhm.201901391. PMC7340246 32329234

[ref32] Truong N. F.; Kurt E.; Tahmizyan N.; Lesher-Pérez S. C.; Chen M.; Darling N. J.; Xi W.; Segura T. Microporous Annealed Particle Hydrogel Stiffness, Void Space Size, and Adhesion Properties Impact Cell Proliferation, Cell Spreading, and Gene Transfer. Acta Biomater. 2019, 94, 160–172. 10.1016/j.actbio.2019.02.054. 31154058 PMC7444265

[ref33] Holt S. E.; Rakoski A.; Jivan F.; Pérez L. M.; Alge D. L. Hydrogel Synthesis and Stabilization via Tetrazine Click-Induced Secondary Interactions. Macromol. Rapid Commun. 2020, 41 (14), 2000287 10.1002/marc.202000287. PMC8085762 32515861

[ref34] Holt S. E.; Arroyo J.; Poux E.; Fricks A.; Agurcia I.; Heintschel M.; Rakoski A.; Alge D. L. Supramolecular Click Product Interactions Induce Dynamic Stiffening of Extracellular Matrix-Mimetic Hydrogels. Biomacromolecules 2021, 22 (7), 3040–3048. 10.1021/acs.biomac.1c00485. 34129338

[ref35] Arkenberg M. R.; Dimmitt N. H.; Johnson H. C.; Koehler K. R.; Lin C.-C. Dynamic Click Hydrogels for Xeno-Free Culture of Induced Pluripotent Stem Cells. Adv. Biosyst. 2020, 4 (11), e2000129 10.1002/adbi.202000129. 32924337 PMC7704730

[ref36] Dimmitt N. H.; Arkenberg M. R.; de Lima Perini M. M.; Li J.; Lin C.-C. Hydrolytically Degradable PEG-Based Inverse Electron Demand Diels–Alder Click Hydrogels. ACS Biomater. Sci. Eng. 2022, 8 (10), 4262–4273. 10.1021/acsbiomaterials.2c00714. 36074814 PMC9554872

[ref37] Dimmitt N. H.; Lin C.-C. Degradable and Multifunctional PEG-Based Hydrogels Formed by IEDDA Click Chemistry with Stable Click-Induced Supramolecular Interactions. Macromolecules 2024, 57 (4), 1556–1568. 10.1021/acs.macromol.3c01855. 38435678 PMC10903513

[ref38] Fairbanks B. D.; Schwartz M. P.; Halevi A. E.; Nuttelman C. R.; Bowman C. N.; Anseth K. S. A Versatile Synthetic Extracellular Matrix Mimic via Thiol-Norbornene Photopolymerization. Adv. Mater. 2009, 21 (48), 5005–5010. 10.1002/adma.200901808. 25377720 PMC4226179

[ref39] Dawson M. E. Depyrogenation. LAL Update 1993, 11 (5), 1–4.

[ref40] Patterson J.; Hubbell J. A. Enhanced Proteolytic Degradation of Molecularly Engineered PEG Hydrogels in Response to MMP-1 and MMP-2. Biomaterials 2010, 31 (30), 7836–7845. 10.1016/j.biomaterials.2010.06.061. 20667588

[ref41] Temenoff J. S.; Athanasiou K. A.; Lebaron R. G.; Mikos A. G. Effect of Poly(Ethylene Glycol) Molecular Weight on Tensile and Swelling Properties of Oligo(Poly(Ethylene Glycol) Fumarate) Hydrogels for Cartilage Tissue Engineering. J. Biomed. Mater. Res. 2002, 59 (3), 429–437. 10.1002/jbm.1259. 11774300

[ref42] Nguyen Q. T.; Hwang Y.; Chen A. C.; Varghese S.; Sah R. L. Cartilage-like Mechanical Properties of Poly (Ethylene Glycol)-Diacrylate Hydrogels. Biomaterials 2012, 33 (28), 6682–6690. 10.1016/j.biomaterials.2012.06.005. 22749448 PMC3572364

[ref43] Lutolf M. P.; Lauer-Fields J. L.; Schmoekel H. G.; Metters A. T.; Weber F. E.; Fields G. B.; Hubbell J. A. Synthetic Matrix Metalloproteinase-Sensitive Hydrogels for the Conduction of Tissue Regeneration: Engineering Cell-Invasion Characteristics. Proc. Natl. Acad. Sci. U.S.A. 2003, 100 (9), 5413–5418. 10.1073/pnas.0737381100. 12686696 PMC154359

[ref44] Anderson S. B.; Lin C.-C.; Kuntzler D. V.; Anseth K. S. The Performance of Human Mesenchymal Stem Cells Encapsulated in Cell-Degradable Polymer-Peptide Hydrogels. Biomaterials 2011, 32 (14), 3564–3574. 10.1016/j.biomaterials.2011.01.064. 21334063 PMC3085912

[ref45] Shin Y. M.; La W.-G.; Lee M. S.; Yang H. S.; Lim Y.-M. Extracellular Matrix-Inspired BMP-2-Delivering Biodegradable Fibrous Particles for Bone Tissue Engineering. J. Mater. Chem. B 2015, 3 (42), 8375–8382. 10.1039/C5TB01310K. 32262890

[ref46] Fan Q.; Bai J.; Shan H.; Fei Z.; Chen H.; Xu J.; Ma Q.; Zhou X.; Wang C. Implantable Blood Clot Loaded with BMP-2 for Regulation of Osteoimmunology and Enhancement of Bone Repair. Bioact. Mater. 2021, 6 (11), 4014–4026. 10.1016/j.bioactmat.2021.04.008. 33997490 PMC8085758

[ref47] Gautschi O. P.; Frey S. P.; Zellweger R. BONE MORPHOGENETIC PROTEINS IN CLINICAL APPLICATIONS. ANZ. J. Surg. 2007, 77 (8), 626–631. 10.1111/j.1445-2197.2007.04175.x. 17635273

[ref48] Krishnakumar G. S.; Roffi A.; Reale D.; Kon E.; Filardo G. Clinical Application of Bone Morphogenetic Proteins for Bone Healing: A Systematic Review. Int. Orthop. 2017, 41 (6), 1073–1083. 10.1007/s00264-017-3471-9. 28424852

[ref49] Azagarsamy M. A.; Anseth K. S. Bioorthogonal Click Chemistry: An Indispensable Tool to Create Multifaceted Cell Culture Scaffolds. ACS Macro Lett. 2013, 2 (1), 5–9. 10.1021/mz300585q. 23336091 PMC3547663

[ref50] Bird R. E.; Lemmel S. A.; Yu X.; Zhou Q. A. Bioorthogonal Chemistry and Its Applications. Bioconjugate Chem. 2021, 32 (12), 2457–2479. 10.1021/acs.bioconjchem.1c00461. 34846126

[ref51] Yang J.; Jacobsen M. T.; Pan H.; Kopecek J. Synthesis and Characterization of Enzymatically Degradable PEG-Based Peptide-Containing Hydrogels. Macromol. Biosci. 2010, 10 (4), 445–454. 10.1002/mabi.200900295. 20146210 PMC4608387

[ref52] Liu Y.; Suarez-Arnedo A.; Riley L.; Miley T.; Xia J.; Segura T. Spatial Confinement Modulates Macrophage Response in Microporous Annealed Particle (MAP) Scaffolds. Adv. Healthcare Mater. 2023, 12 (26), 2300823 10.1002/adhm.202300823. PMC10592513 37165945

[ref53] Pfaff B. N.; Flanagan C. C.; Griffin D. R. Microporous Annealed Particle (MAP) Scaffold Pore Size Influences Mesenchymal Stem Cell Metabolism and Proliferation Without Changing CD73, CD90, and CD105 Expression Over Two Weeks. Adv. Biol. 2024, 8 (2), e2300482 10.1002/adbi.202300482. PMC10922193 37955859

[ref54] Karver M. R.; Weissleder R.; Hilderbrand S. A. Synthesis and Evaluation of a Series of 1,2,4,5-Tetrazines for Bioorthogonal Conjugation. Bioconjugate Chem. 2011, 22 (11), 2263–2270. 10.1021/bc200295y. PMC3254257 21950520

