# Supplementary material for: Impact of Annealing Chemistry on the Properties and Performance of Microporous Annealed Particle Hydrogels
Source: Biomacromolecules. 2024 Aug 27;25(9):5798–808. doi: 10.1021/acs.biomac.4c00465 (PMC11388458; doi:10.1021/acs.biomac.4c00465)
Supplement: Supplementary file 1 — bm4c00465_si_001.pdf [file bm4c00465_si_001.pdf]

SUPPORTING INFORMATION

Impact of Annealing Chemistry on the Properties and Performance of Microporous Annealed Particle Hydrogels

Sarea Y. Recalde Phillips[1], Kiara D. Perez-Ponce[1], Elizabeth Ruben[1], Talia Baig[1], Emily Poux[2], Carl A. Gregory[1,3], Daniel L. Alge[1,4*]

[1]Department of Biomedical Engineering, Texas A&M University, College Station, TX 77843, United States
[2]Department of Chemical Engineering, Texas A&M University, College Station, TX 77843, United States
[3]Department of Cell Biology and Genetics, School of Medicine, Texas A&M University, Bryan, TX 77807, United States
[4]Department of Materials Science and Engineering, Texas A&M University, College Station, TX 77843, United States

*Email: dalge@tamu.edu

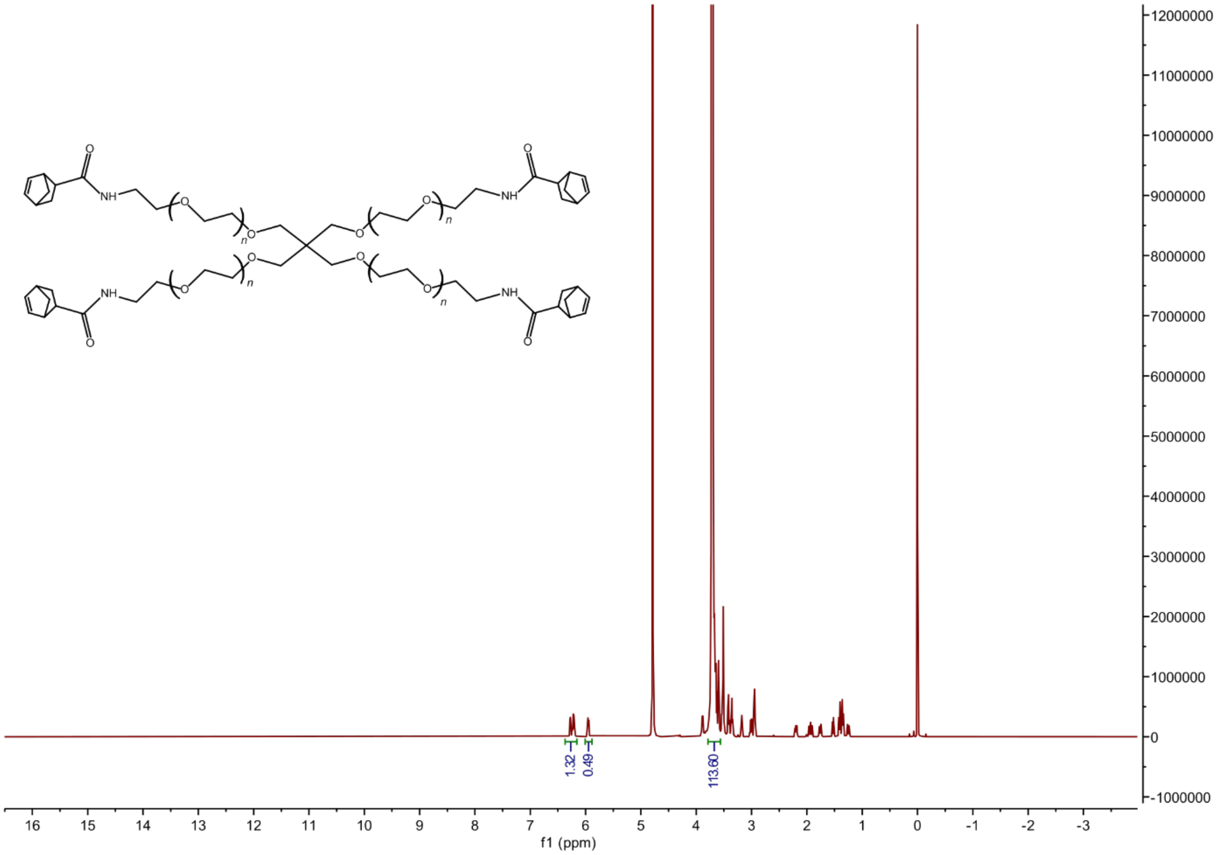

Figure S1. ^{1}H NMR spectrum of norbornene-functionalized PEG (MW = 5kDa). PEG was dissolved in D_2O, and functionalization was calculated by normalizing the alkene protons from the norbornene end group (δ = 6.22 – 6.06) to the PEG (δ = 3.7 – 3.6).

Microgel Type	PEG-aNB [mM]	KCGPQGIWGQCK [mM]	CGRGDS [mM]	LAP [mM]
5kDa	18.25	24.27	1	10
20 kDa	4.88	6.82	1	10

Table S1. Microgel formulation used during electrospraying. A thiol:norbornene ratio of 0.75:1 was maintained across microgel formulations to ensure that excess norbornenes would be available for annealing of TzMAP and ThiolMAP hydrogels.

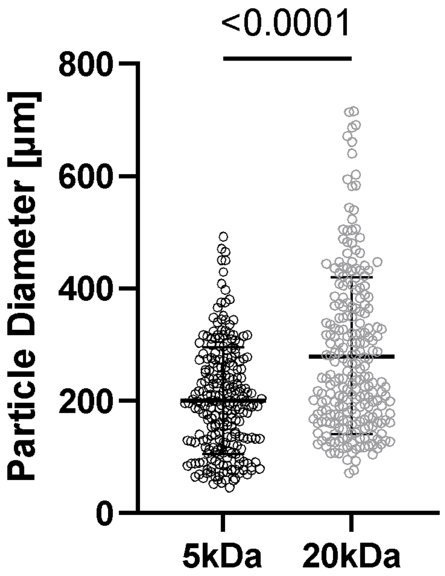

Figure S2. Diameter of 5kDa and 20kDa microgels used for MAP hydrogel assembly. Data is represented as mean ± standard deviation. n = 234.

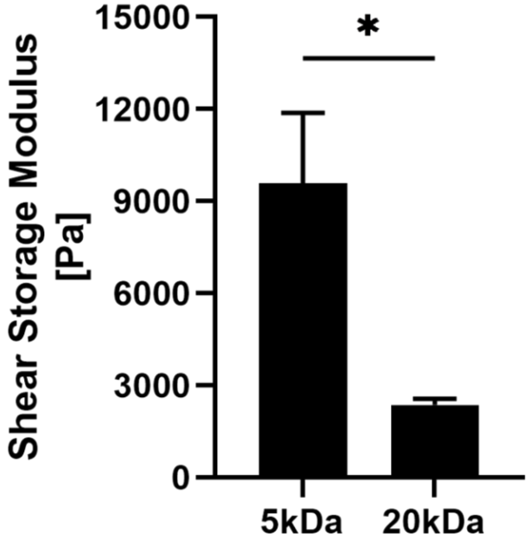

Figure S3. Shear storage modulus of 5kDa and 20kDa bulk hydrogels. Hydrogels were formed using the same formulations as were used for microparticle synthesis. Specifically, 10wt% PEG pre-polymer solutions containing a 0.75:1 thiol:norbornene ratio were irradiated in 8mm-diameter molds for 5min at 60mW/cm^2. Hydrogels were swelled overnight in PBS prior to rheology. Data is represented as mean ± standard deviation. *p < 0.05. n = 3 hydrogels.

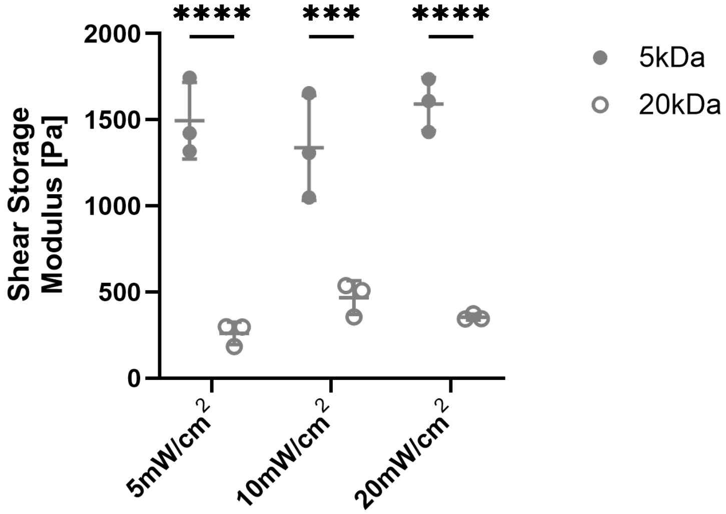

Figure S4. Shear storage modulus of 5kDa and 20kDa ThiolMAP hydrogels assembled with varying light intensity. Data is represented as mean ± standard deviation. **** = p < 0.0001. n = 3 hydrogels.

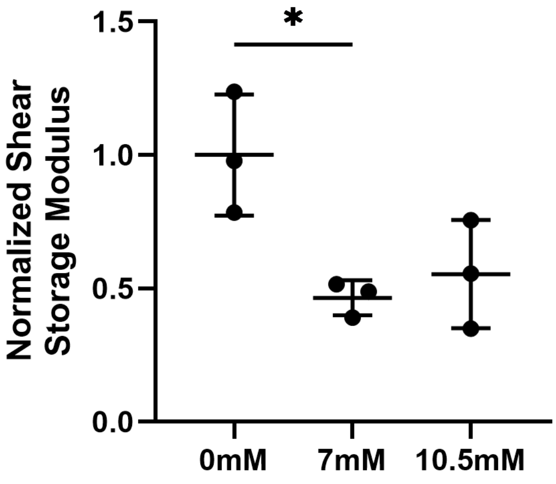

Figure S5. Impact of available norbornene groups on TzMAP shear storage modulus. Unreacted norbornene groups on 5 kDa microgels were capped through photopolymerization at 10mW/cm^2 in 1mM LAP and varying concentrations of L-cysteine. Microgels were subsequently used to anneal TzMAP hydrogels with PEG-di-Tz. Data were normalized to the average modulus of the uncapped group and are represented as mean ± standard deviation. * = $p < 0.05$. n = 3 hydrogels.

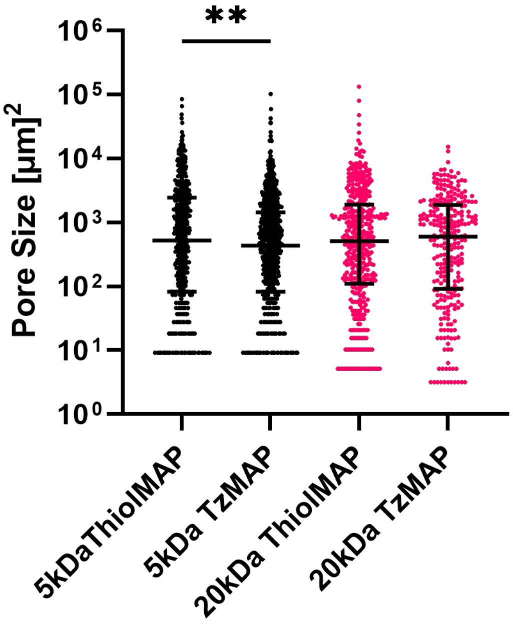

Figure S6. Distribution of pore size area in 5kDa and 20kDa MAP hydrogels. Data points represent individual pores, and the error bars represent the median and interquartile ranges. **$p < 0.01$. n = 3 hydrogels.

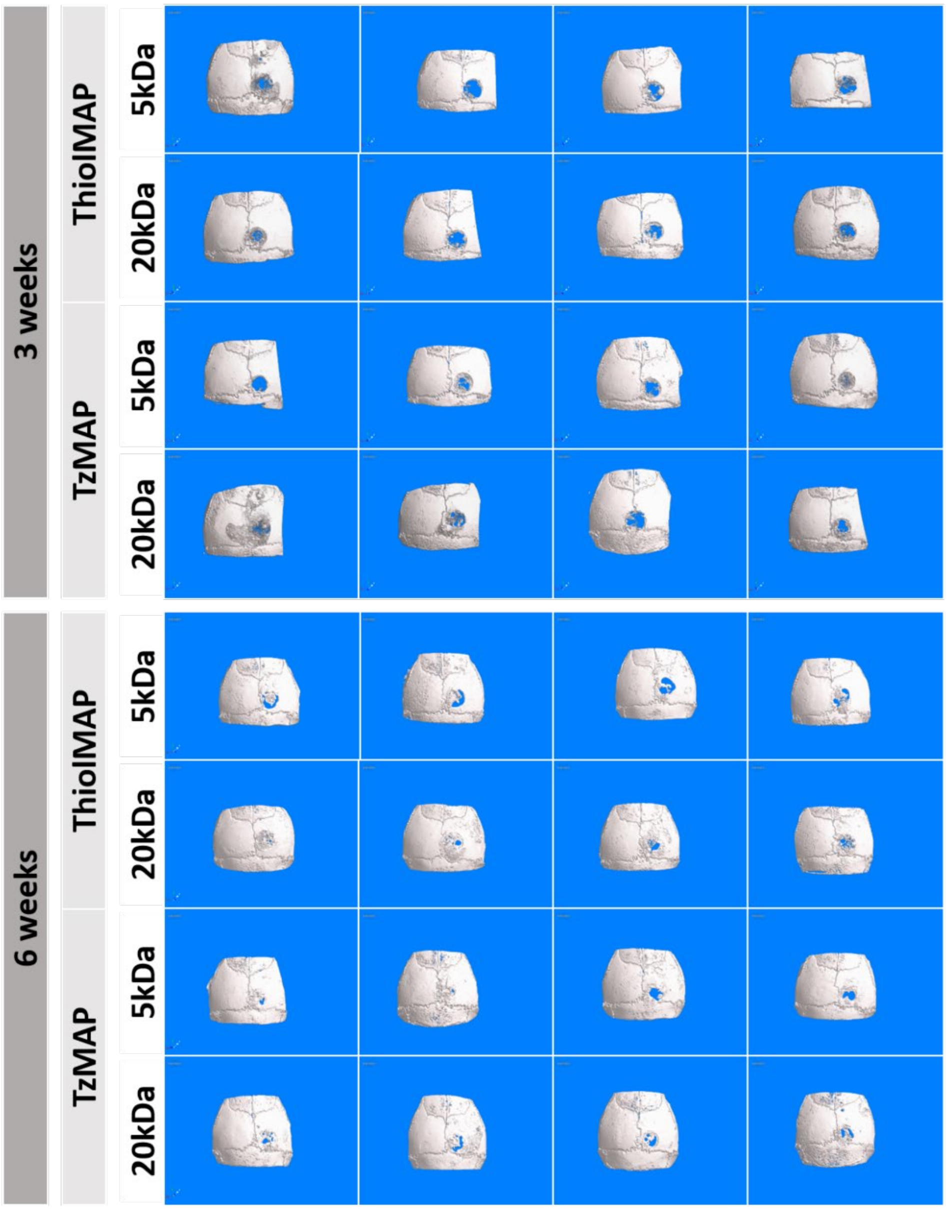

Figure S7. 3D reconstructions of calvarial samples 3 and 6 weeks post-implantation of TzMAP and ThiolMAP hydrogels. Images were generated from scans obtained from the Skyscan instrument. Displayed reconstructions represent the calvarium of each mouse used during the study.

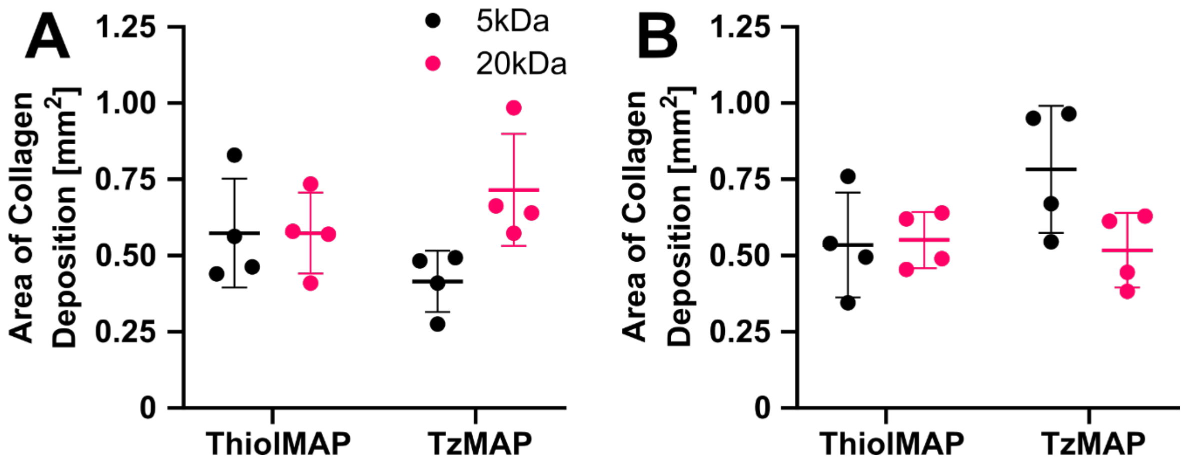

Figure S8. Collagen deposition at defect sites (A) 3 weeks and (B) 6 weeks post-implantation of TzMAP and ThiolMAP hydrogels. Data is represented as mean ± standard deviation. n = 4.
